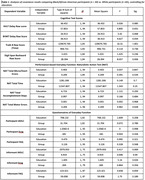# Assessing Race Differences on Performance‐Based Assessment in Older Adults

**DOI:** 10.1002/alz.092697

**Published:** 2025-01-09

**Authors:** Marina Kaplan, Moira Mckniff, Stephanie M. Simone, Anna Callahan, Tania Giovannetti

**Affiliations:** ^1^ Temple University, Philadelphia, PA USA

## Abstract

**Background:**

Race differences on cognitive tests have been widely reported and attributed to psychosocial factors. Cognitive assessment via performance‐based tests of everyday functioning may be less influenced by psychosocial factors, offering ecologically valid and less/unbiased measurement. This study examined differences between Black/African American and White older adults on traditional cognitive tests, self/informant report of everyday function, and a sensitive performance‐based test of everyday function called the Naturalistic Action Task (NAT). We predicted group differences on only traditional cognitive tests.

**Method:**

100 community dwelling older adults (M age = 73±6.88; 66% Female; 65% White; M education = 16±2.69) from the Philadelphia area completed cognitive tests of episodic memory and executive functioning as well as the NAT, which requires making a breakfast and a lunch using objects on a table. Video recordings of NAT performance were scored for cognitive aspects of performance (accomplishment of task steps, completion time, and errors) as well motor imprecisions/clumsiness (motor errors). Participants and informants also completed questionnaires of the participants’ everyday functioning (Functional Activity Questionnaire, Measurement of Everyday Cognition, The Instrumental Activities of Daily Living – Compensation). Analyses of Covariance adjusting for education were used to evaluate group differences.

**Result:**

The groups differed in education level but not age or sex distribution. As shown in Table 1, after controlling for education, the groups differed on tests of episodic memory and executive function. By contrast, there were no group differences on the NAT cognitive measures, though Black/African American participants made significantly more motor errors. Finally, there were no group differences on any of the self/informant questionnaires.

**Conclusion:**

Consistent with prior literature, Black/African American older adults and White older adults differed significantly on traditional cognitive tests, even after controlling for education. Group differences were not observed on questionnaires nor on cognitive measures from the performance‐based test of everyday functioning (NAT). However, contrary to prediction Black/African American participants made more motor errors on the performance‐based test (NAT). In sum, performance‐based tests, such as the NAT, should be considered as an alternative to traditional cognitive tests in research and clinical contexts that include racially diverse older adults.